# Filtering the Signal: Kappa Opioid Receptors in the Kidney

**DOI:** 10.1093/function/zqaf042

**Published:** 2025-09-03

**Authors:** Mohammad O Sako, Nirupama Ramkumar

**Affiliations:** Department of Internal Medicine, Division of Nephrology and Hypertension, University of Utah, Salt Lake City, UT 84132, USA; Department of Internal Medicine, Division of Nephrology and Hypertension, University of Utah, Salt Lake City, UT 84132, USA; Diabetes and Metabolism Research Center, University of Utah, Salt Lake City, UT 84112, USA

## A Perspective on “Renal Implications of Kappa Opioid Receptor Signaling in Sprague Dawley Rats”

Opioids are one of the oldest class of medications used in human history dating back to 3500 bce for its analgesic and euphoric properties. Opioids exert their effects through four main opioid receptors namely delta (DOR), Kappa (KOR), mu opioid receptor (MOR), and nociception/orphanin FQ receptor (NOPR), all of which are G protein coupled receptors.^1^ Although opioid receptor expression is predominantly in the central and peripheral nervous system, other organs such as the kidney, heart, gastrointestinal tract, adrenal gland, and immune cells also express opioid receptors with the expression pattern varying across receptor types and mammalian species. Within the kidney, KOR expression has been described consistently in humans and rodents, while the expression of the other opioids receptors remains conflicting.^2^ Besides analgesia, KOR activation in the kidney has been linked to diuresis and natriuresis,^3^ blood pressure and renoprotection against ischemia/reperfusion injury.^4^

In this issue of *Function*, Didik et al.^5^ delve into the role of KOR signaling in kidney podocytes in Sprague-Dawley rats using a selective agonist, BRL 52537. Acute BRL 52537 treatment in podocytes isolated from SD rats demonstrated increased calcium influx along with disruption of the glomerular filtration barrier in freshly isolated glomeruli. Further, intravenous administration of BRL 52537 daily for 2 wk caused a sustained increase in mean arterial pressure (MAP) of approximately 10 mm of Hg. Concomitant increase in albuminuria was observed at day 14 of treatment without change in medullary protein casts or cortical interstitial fibrosis, indicating that the elevated MAP and albuminuria were reflective of podocyte damage. There was, however, a modest increase in glomerular injury score with BRL 52537 treatment and podocytes isolated from SD treated for 14 days also demonstrated higher basal intracellular calcium levels. These findings build upon the group’s previous work^6^ where they investigated the effects of BRL 52537 treatment in Dahl SS rats. Consistent with the current study, BRL 52537 mediated calcium influx in cultured human podocytes and freshly isolated rat glomeruli with changes in podocyte cytoskeleton leading to reduced cell surface area. Furthermore, chronic KOR agonism in Dahl SS rats induced podocyte damage, albuminuria, and hypertension following salt challenge. Interestingly, BRL 52537 effects on podocytes in the latter study were found to be mediated via Transient Receptor Potential Canonical 6 (TRPC6) channel which was not examined in the current study. Further, there was no evidence of overt podocyte injury in Didik’s study as demonstrated by unchanged expression of podocin or nephrin in the glomeruli. While this could be partly explained by the additive deleterious effects of salt and KOR agonism on podocytes, further studies will help inform the precise molecular signaling pathways of KOR activation in podocytes downstream of enhanced calcium influx.

Another key finding from Didik’s study is that BRL 52537 treatment induced diuresis without natriuresis or kaliuresis starting from day 1 of treatment. These results were similar to a recent study by Meariman et al., which showed that nalfurafine, a KOR agonist, enhanced diuresis but reduced sodium and potassium excretion^3^ with onset of effect as soon as 20 min post injection and persisting throughout the 90-min duration of the experiment.^3^ Notably, there was a significant decline in MAP following a single IV injection in SD rats in the Meariman study suggesting that the acute vs chronic effects of KOR activation might be opposing.

To elaborate on the potential underlying mechanisms of elevated MAP, Didik et al. examined plasma levels of the renin-angiotensin-aldosterone system as well as endothelin levels in the kidney cortex. Both systems were unaffected by BRL 52537 treatment implying that other regulatory systems might be involved. Previous studies have reported a biphasic effect of KOR receptor agonist BRL 52656 on blood pressure mainly as these agonists can cross the blood brain barrier.^7^ Low doses of BRL 52656 decreased blood pressure, while high doses produced the opposite effect in spontaneously hypertensive rats.^7^ Given that BRL 52537, like 52656 can cross the BBB,^8^ it is possible that activation of the sympathetic nervous system might alter glomerular hemodynamics and explain the elevated blood pressure seen in Didik’s study.

Given the widespread use of opioids in clinical practice, particularly since opioids are preferred pain relievers in patients with chronic kidney disease, comprehensive assessment of opioids on kidney function and blood pressure is acutely warranted. Didik’s study is timely and informative in delineating the molecular and physiological effects of chronic KOR agonism on podocyte health and function. Their findings highlight the complexity of opioid receptor signaling within the kidney and illustrate the context and dose dependent regulation of blood pressure. Moreover, opioid receptor activation/inhibition appears to have significant impact based on the disease model used, the dose and physiochemical properties of the drug. For instance, MOR antagonism by naloxone was shown to be protective against ischemia-reperfusion injury while morphine, a non-selective opioid receptor agonist was linked to podocyte injury in otherwise healthy mice.^9,10^ Clearly, further investigation is needed to identify the long term (more than 14 days) effects, the downstream mechanisms of KOR mediated blood pressure changes and kidney injury as well as potential interaction of KOR with other opioid receptors within the kidney. This would provide a clear identification of both the potential groups of patients more susceptible to developing subsequent renal injury and agents/doses to be provided or excluded.
